# Sputum smear conversion and associated factors among smear-positive pulmonary tuberculosis patients in East Gojjam Zone, Northwest Ethiopia: a longitudinal study

**DOI:** 10.1186/s12890-021-01483-w

**Published:** 2021-04-08

**Authors:** Mulusew Andualem Asemahagn

**Affiliations:** School of Public Health, College of Medicine and Health Sciences, Bahir Dar University, Bahir Dar, Ethiopia

**Keywords:** Pulmonary TB, Smear conversion, Factors, East Gojjam, Ethiopia

## Abstract

**Background:**

Sputum smear conversion is a key indicator of treatment response and reduced infectivity among bacteriologically confirmed pulmonary tuberculosis (PTB) patients. This study aimed at estimating sputum smear conversion and identifying factors hindering sputum smear conversion among bacteriologically confirmed PTB cases in East Gojjam Zone, Northwest Ethiopia.

**Methods:**

A total of 282 bacteriologically confirmed PTB patients were followed for 22 weeks through weekly sputum smear examination. Due to the absence of sputum culture and rapid diagnostic services, sputum smear conversion evaluation was conducted microscopically using acid-fast-bacilli staining technique of sediments from a 5% sodium hypochlorite concentration technique. Data on socio-demographic, clinical profile and personal behavior variables were collected using a pretested interviewer-administered questionnaire. Various descriptive statistics including mean, median with interquartile range (IQR), and proportions were computed to describe study objectives. Factors of sputum smear conversion were identified by multivariable logistic regression analysis and statistical significance was determined at a *p* value < 0.05.

**Results:**

Over half, 166 (59%) of bacteriologically confirmed PTB patients were males and 147 (52%) were rural dwellers. The mean age of respondents was 35 ± 5 SD years. About 88 (31.2%) of bacteriologically confirmed PTB patients had comorbidities, 102 (36.2%) faced stigma, and 54 (19%) history of cigarette smoking. The median sputum smear conversions during the intensive phase and 5th  months of treatment follow up were 35 dyas (IQR: 21-56 days) and 53 days (IQR: 28-82 days), respectuvely. The majority, 85% (95% CI 76–93%) and 95% (95% CI 85–99%) of bacteriologically confirmed PTB patients underwent sputum smear conversion at the end of 2nd and 5th months of treatment, respectively. Poor knowledge on TB, being HIV positive, higher smear grading, having diabetes mellitus, undernutrition, cigarette smoking, facing societal stigma, and TB service delays were positively associated with the length of sputum smear conversion (*p* value < 0.05).

**Conclusion:**

Based on this study, the median sputum smear conversion time was higher compared to TB program expectations and findings from former studies. The study also identified important factors associated with sputum smear conversion time. Improving health literacy of the community by revising the existing community awareness strategies is essential to enhance treatment adherence and lower infectiousness after treatment initiation.

**Supplementary Information:**

The online version contains supplementary material available at 10.1186/s12890-021-01483-w.

## Introduction

Several millions of lives have been saved because of various global and national anti-TB interventions [1]. Tuberculosis, largely caused by mycobacterium tuberculosis, however, remains a global health problem with varying magnitudes across countries and regions [1, 2]. Countries with poor socio-economic conditions have been affected more by TB in terms of morbidity, mortality, and cost for care [2, 3]. As a result, over 10 million new TB cases were reported in 2020 where 88% of those  cases were among people aged ≥ 15 years. Over half, 56% was among adult males and there were about 1.4 million TB deaths. About 44% of the global new TB cases were from South-East-Asian countries followed by 25% from African countries. South Africa and Nigeria are among the top eight high TB burden countries [1].

Among 14 high TB, TB-HIV, and multi-drug resistant TB (MDR-TB) countries, eight are from Africa including Ethiopia. High HIV burdens [3–5], poor access and quality of healthcare services [3, 6, 7], poverty [1, 7], and high population growth and mobility [1, 3, 8] are major factors associated with the ongoing TB burdens in developing countries. TB is among the top ten public health problems in Ethiopia with varying magnitudes across regions, zones and districts [9, 10]. TB is a problem of both urban and rural populations in Ethiopia [11, 12]. Poor accessibility of TB diagnostic and treatment services [9, 13, 14], poverty [9, 14, 15], high HIV burden [4, 10, 16], low community awareness [15, 17], and poor TB program monitoring and supports [7, 15] have contributed more to the presence of high TB burdens in Ethiopia.

Early case detection and treatment of the detected cases are the most effective strategies of TB control programs [1]. However, their performance is very far from the expected level globally and worse in high TB burden countries [1, 12, 15]. As a result, more TB cases remain undetected, detected cases did not get full service of anti-TB treatment, and developing countries are suffering from poor treatment outcomes that lead to MDR-TB [1, 3, 11, 18]. The global TB control programs have two phases of anti-TB treatment through sputum smear follow-up at end of 2nd months treatment (the intensive phase) and end of the 5th-month treatment [19]. This is to check the effectiveness of anti-TB treatment follow-up, take action on anti-TB-treatment to follow up, and know the status of treatment outcome of each TB patient [20]. Although most TB cases are expected to become smear-negative after taking drugs of the intensive phase treatment, a significant number of PTB cases remain bacteriologically positive [20]. This increases the length of the infectious period of PTB cases [21].

Most of the developing countries lacked information on time of  smear conversion due to poor documentation, absence of TB diagnostic tools, and poor TB program monitoring strategies [18, 20, 21]. Similarly, identifying determinant factors that resulted in delayed sputum smear conversion is also vital to improve the effectiveness of TB treatment follow-up [20, 22, 23]. The situation has no exception to the Ethiopian TB prevention and control programs [21]. Little is known about the time of  sputum smear conversion among bacteriologically confirmed PTB cases. Although there are few studies, they are among the MDR-TB patients [23]. In addition, we are not aware of factors that lengthen the sputum smear conversion.

Therefore, we conducted a study on sputum smear conversion and associated factors among bacteriologically confirmed PTB patients in East Gojjam zone, Northwest Ethiopia. The reason for choosing this zone is due to its high TB burdens [21, 24], lowest TB case detection [24], and no former studies on sputum smear conversion among newly diagnosed PTB patients. Thus, study findings will be important evidence to districts, zonal and regional level TB control programs to make informed decisions to control factors and improve the effectiveness of anti-TB treatment follow-up period. It will also be relevant literature to the coming researchers in TB prevention and control.

## Materials and methods

### Study design and settings

A facility-based longitudinal study was conducted between January-June 2019 among 282 new bacteriologically confirmed PTB patients in East Gojjam Zone of the Amhara Region, Northwest Ethiopia. East Gojjam zone, one of the 12 Zones in the Amhara Regional State, has a total estimated population of 2,740,625 in 2019. About 85% of the zonal population is living in rural areas. The zone has about 14,010 km^2^ area coverage divided into 19 administrative woredas (a third-level administrative hierarchy in Ethiopia). The zone has about 517 public health facilities (406 health posts/HPs/, 102 health centers/HCs/, and nine hospitals) with a history of offering TB services at the time of data collection. Sputum smear microscopy is a principal TB diagnostic tool in all health facilities of the zone [13, 24].

### Source and study population

All the newly diagnosed bacteriologically confirmed PTB patients at the first day and first week of their intensive phase of anti-TB treatment were the source population to this study. Whereas, all the adult PTB cases (aged ≥ 15 years) at the first day and first week of their intensive phase of anti-TB treatment in the selected health facilities were the study population. The reason for excluding PTB cases aged < 15 years was related to their physiology and ability to offer purulent sputum for sputum smear evaluation  up to 22 weeks. Usually, children under 15 years have a problem of giving purulent sputum and most are extrapulmonary and smear-negative [1, 25]. If we included under 15 years children, it might have an impact on the estimation of sputum smear conversion. PTB cases who were seriously ill and unable to understand and respond to questions were also excluded from being study samples.

### Sample size determination and sampling techniques

The sample size (282) was calculated by Epi Info version 7 using a total of 763 smear-positive PTB cases from the study area in 2019 [24], 95% confidence interval, 5% margin of error, 50% proportion of sputum smear conversion proportion among PTB patients since no former published study on sputum smear conversion and associated factors among newly diagnosed bacteriologically confirmed PTB patients. A 10% non-response rate was also considered while estimating sample size. To select study health facilities, we considered the rule of thumb and resource constraints to include 25% of health facilities offering TB services (102 HCs and nine hospitals). According to the Ethiopian healthcare delivery system [26], one health center and five HPs around that HC are termed as primary healthcare unit (PHCU). Based on that, 158 health facilities (two hospitals, 26 HCs, and 130 health posts) were included in the study through the simple random (computer random number generation) technique. Accordingly, we included 26 PHCUs and two hospitals. The sample size was allocated to those hospitals and PHCUs proportionally based on the number of smear-positive PTB cases they had during the data collection period. Then, all the new bacteriologically confirmed PTB patients in their first week of the intensive phase were considered until getting the required sample size.

### Data collection tools and procedures

Data on socio-demographic, clinical profile and personal behavior-related variables of bacteriologically confirmed PTB patients were collected using an interviewer-administered questionnaire (Additional file 2). The questionnaire was developed by referring to former similar studies on factors of sputum conversion [20, 22, 23]. The questionnaire was pretested among bacteriologically confirmed PTB patients from health facilities outside of the study area but having the same characteristics. Necessary modifications on order, clarity, relevance, and completeness of questions were made based on results from a pretest. Six trained clinical nurses and two public health supervisors collected data through a face-to-face interview of each smear-positive PTB patient. Data were collected after getting written informed consent from each study participant.

### Sputum sample collection and processing

A morning sputum specimen was collected from each newly diagnosed bacteriologically confirmed PTB patient weekly up to 22 weeks follow-up period. Three trained laboratory technologists collected and processed the sputum specimens following the Ethiopian national TB diagnosis and treatment guideline adapted from the WHO guideline [8, 16]. Due to the absence of sputum culture and rapid TB diagnostic services in the health facilities of the study area, sputum smears were processed by Ziehl–Neelsen acid-fast bacilli (AFB) staining technique and examined for the presence of bacilli microscopically. A sputum concentration technique using a 5% sodium hypochlorite solution was used to process sputum samples to increase the concentration and detection of bacilli. We used labeled sputum cap, applicator stick, 5% sodium hypochlorite, distilled water, centrifuged, AFB reagents, plastic bulb pipette, slide, cover slide, immersion oil, and Olympus microscope to process sputum specimens. A 2 ml sputum sample was transferred to a test tube of 15–20 ml volume. An equal volume of sodium hypochlorite (bleach) solution was added to a test tube that contains a 2 ml sputum sample and mixed well. Then the mixture was left for 20–30 min at room temperature through frequent shaking to break down the mucus in the sputum. Afterward, 8 ml distilled water was added to the mixture, mixed well, and centrifuged at 3000 rpm for 15 min. Lastly, the supernatant fluid was removed using a plastic bulb pipette and the smear was prepared from the sediment through the Ziehl Neelsen AFB staining technique [16, 27]. The three trained laboratory technologists examined all prepared smears turn by turn using an Olympus microscope and final results were reported after the approval of three technologists.

### Operational definitions

#### Sputum smear conversion

PTB patients were reported as sputum smears converted when the reading of sputum smears became negative after a certain period of anti-TB treatment. It was measured using median time sputum smear conversion and IQR.

#### Delayed sputum smear conversion

PTB patients were reported as having delayed sputum smear conversion if their sputum smear conversions were beyond the median sputum smear conversion (> 35 days).

#### Favorable treatment outcome

PTB patients were grouped as having favorable treatment outcomes if their treatment outcomes were reported as cured and completed (cured + treatment completed).

#### Knowledge on TB

The knowledge of each bacteriologically confirmed PTB patient was measured using 12 knowledge questions. PTB patients who scored above the mean score of 12 knowledge questions were grouped as having good knowledge about TB.

#### Faced stigma

A PTB patient was reported as faced stigma if he/she got at least one unusual reaction/sign from their relatives, families, friend, and the community after they knew the TB status of him/her.

#### Length of TB service delay

It was measured by calculating the total delayed days in terms of median time from the first onset of cough to the date of anti-TB treatment initiation (the sum of median delayed days of PTB patients, diagnosis, and treatment service).

### Data quality assurance

Providing 2 days of training for data collectors, pretesting of a questionnaire, regular supportive supervision of data collectors, double data entry, labeling sputum samples and smear slides with patient’s identification number, checking data completeness, and processing sputum samples according to the national and WHO TB diagnosis and treatment guidelines were data quality control activities. In addition, data collectors and supervisors used a data collection guide book, and the result of each sputum smear was reported after being examined by three laboratory technologists orderly.

### Data processing and analysis

Data were entered, cleaned, and analyzed using SPSS version 25. Various types of descriptive statistics such as mean, median, interquartile range, proportions, and cross-tabulations were computed to describe study variables. Anti-TB treatment outcome was measured in terms of favorable (cured) and unfavorable (failed). Each processed sputum smear was examined and reported for each bacteriologically confirmed PTB patient for 22 weeks duration. The bacteriologically confirmed PTB patients who had a history of cigarette smoking of any type, dose, and frequency during anti-TB treatment period were considered smokers. Similarly, bacteriologically confirmed PTB patients who had a history of taking any type of alcohol and amount during anti-TB treatment period were considered alcohol drinkers. Moreover, bacteriologically confirmed PTB patients who had chewed chat with any type, dose, and frequency during treatment follow up period were grouped as chat chewers. The reasons for considering any type, amount (dose), and duration for personal behaviors were just by assuming these substances  are risky for TB patients on anti-TB treatment in the form of drug-substance reactions, discomforts, forget to take anti-TB drugs and appointments (check-up and collecting anti-TB drugs). The sputum smear conversion time was measured using median time with IQR and proportions (Additional file 1). Nutritional status of PTB patients was measured in terms of Body mass index (BMI). PTB patients who had a BMI of ≤ 18.5 kg/m^2^ were considered as undernutrition and those who had > 18.5 kg/m^2^ were grouped as normal since normal and overweight nutritional status have inversely association with TB infection [16]. Model fitness was checked using Hosmer and Lemeshow test (*p* value > 0.05). Factors associated with sputum smear conversion were identified using bivariate logistic regression analysis. Multivariable logistic regression analysis was used to control the confounding effects. The results of regression associations were described using odds ratio at 95% confidence interval (CI) and variables were considered as statistically significant if they have *p* values < 0.05.

### Ethical clearance

The study was conducted according to the Declaration of Helsinki and fulfilled the Ethiopian National Health Research and Ethics Guideline. The study was conducted after taking ethical clearance and approval letter from the ethical review committee of the College of Medicine and Health Sciences, Bahir Dar University (Protocol No: 091/18-04). Data were collected after getting written informed consent from each PTB patient. Participation was fully voluntary based including the right to withdraw from the study at any time without presenting an explanation for their withdraw. Data confidentiality was maintained through anonymity by avoiding any personal identifiers. The Amhara Regional Health Bureau and in-line offices gave supporting letters after being informed about research objectives and procedures.

## Results

### Socio-demographic and personal behaviors of study participants

A total of 282 newly diagnosed active bacteriologically confirmed PTB cases, 166 (59%) males and 147 (52%) rural dwellers were followed for up to 22 weeks through sputum smear examination. The mean age of respondents was 35 ± 5 SD years. Of 282 PTB patients,  104 (37%) cannot read and write, 119 (42%) had good knowledge on TB and 42 (15%) were HIV positive. Treatment success rate (cured) since we followed up to the end of the 5th-month treatment was 95%. Only 54 (19%) of PTB cases had ≤ 14 days of cough duration before being diagnosed as bacteriologically confirmed PTB. A significant number of bacteriologically confirmed PTB cases (38%) faced societal stigma and discrimination from families, friends, and the community. A large number of bacteriologically confirmed PTB cases, 124 (44%) had greater than or equal to the median TB service delay (≥ 82 days). Over two-thirds, 198 (70%) of the respondents had BMI  values indicating undernutrition (Table 1).

**Table 1:** Socio-demographic, clinical profile, and personal behaviors of smear-positive PTB patients in East Gojjam Zone, Northwest Ethiopia, 2019 (n = 282)

Variable	Response	Number (%)
Age in years	15–35	127 (45.0)
	> 35	155 (55.0)
Sex	Male	166 (59.0)
	Female	116 (41.0)
Residence	Rural	147 (52.0)
	Urban	135 (48.0)
Education level	Cannot read and write	104 (37%)
	Can read and write	178 (63.0)
Previous TB history	Yes	57 (20.0)
	No	225 (80.0)
Marital status	Single	147 (52.0)
	In union	135 (48.0)
Knowledge about TB	Good	119 (42.0)
	Poor	163 (58.0)
Grade/bacilli load	< 2+	175 (62.0)
	≥ 2+	107 (38.0)
HIV status	Positive	42 (15.0)
	Negative	240 (85.0)
Treatment outcome	Favorable	268 (95.0)
	Unfavorable	14 (5.0)
Had diabetes mellitus	Yes	51 (18.0)
	No	231 (82.0)
Had a history of smoking	Yes	54 (20.0)
	No	214 (80.0)
Had a history of chat chewing	Yes	28 (10.0)
	No	254 (90.0)
Had a history of taking alcohol	Yes	73 (26.0)
	No	209 (74.0)
Duration of cough	≤ 14 days	54 (19.0)
	> 14 days	228 (81.0)
Faced societal stigma	Yes	107 (38.0)
	No	175 (62.0)
Body mass index (BMI)	Under nutrition	198 (70.0)
	Normal	84 (30.0)
Length of TB service delay	< 82 days	158 (56.0)
	≥ 82 days	124 (44.0)

### The proportion of sputum smear conversion of PTB cases

**Fig. 1:**
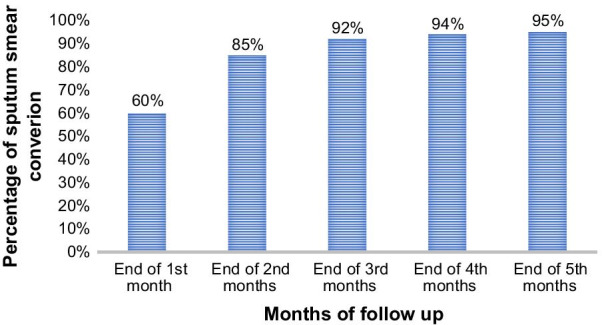
Proportion of sputum smear conversion at the end of 1st, 2nd, 3rd, 4th, and 5th months anti-TB treatment follow up in East Gojjam Zone, Northwest Ethiopia, 2019

The proportions of sputum smear conversion at the end of 1st, 2nd, 3rd, 4th, and 5th months of anti-TB treatment follow up periods were 60% (95% CI 52–72%), 85% (95% CI 76–92%), 92% (95% CI 80–96), 94% (95% CI 85–98), and 95% (95% CI 85–99%), respectively (Fig. 1).

**Fig. 2:**
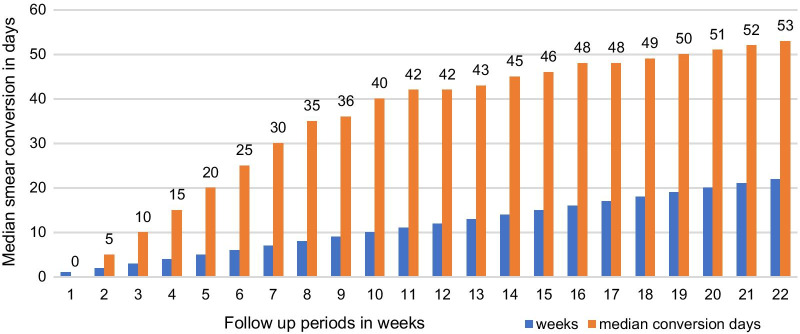
The trend of the time to sputum smear conversion over 22 weeks follow-up period, 2019

Based on the anti-TB treatment follow-up of each PTB case for 22 consecutive weeks, the median sputum smear conversion times during the intensive and follow up periods were found to be 35 days, IQR: 21–56 days, and 53 days, IQR: 28–82 days, respectively (Fig. 2).

### Factors associated with sputum smear conversion

The multivariable logistic regression analysis indicated that BMI, smear grading, HIV infection, having DM, knowledge on TB, cigarette smoking, faced societal stigma, and length of TB service delay were statistically significant factors (*p* value < 0.05) of smear conversion in the study area. Accordingly, the odds of having higher sputum conversion was double times among PTB cases with undernutrition status compared to PTB cases with normal and overweight status (AOR = 2.01, 95% CI = 1.31–3.85). PTB cases having initial smear grading of < 2+ (1+ and scanty) were 54% times less likely to have delayed sputum smear conversion compared to PTB cases with ≥ 2+ sputum smear grading (AOR = 0.46, 95% CI = 0.21–0.73). In addition, the odd of having delayed sputum smear conversion was twice among PTB cases who had HIV compared to HIV-negative PTB cases (AOR = 2.38, 95% CI = 1.20–4.91). Similarly, PTB cases with DM positive status were twice likely to have delayed sputum smear conversion compared to the counterpart PTB cases (AOR = 2.11, 95% CI = 1.18–4.20).

Moreover, PTB cases with cigarette smoking history had a double odd ratio to have delayed sputum smear conversion compared to PTB cases with no history of cigarette smoking (AOR = 1.96, 95% CI = 1.20–3.82). PTB cases who faced societal stigma due to their TB status had AOR = 2.0, 95% CI = 1.26–3.52 times odd to have delayed sputum smear conversion than their counterpart PTB patients. On the other hand, TB service delay showed an inverse association with sputum smear conversion status. Based on that, the odds of having delayed sputum smear conversion was 65% times less likely among PTB patients with low TB service delay compared to PTB patients who had longer TB service delay; AOR = 0.35, 95% CI = 0.23–0.64 (Table 2).

**Table 2:** Factors associated with sputum smear conversion in East Gojjam zone, Ethiopia, 2019 (n = 268)

Variable	Response	Smear conversion time		COR (95% CI)	AOR (95% CI)
		> 35 days	≤ 35 days		
Age in years	15–35	60 (22.4)	67 (25.0)	0.96 (0.59–1.55)	0.68 (0.35–1.42)
	> 35	68 (25.4)	73 (27.2)	1	1
Sex	Male	75 (28.0)	83 (31.0)	0.97 (0.60–1.58)	1.35 (0.26–2.15)
	Female	53 (19.7)	57 (21.3)	1	1
Residence	Rural	62 (23.0)	78 (29.0)	0.75 (0.46–1.23)	0.41 (0.15–1.08)
	Urban	66 (25.0)	62 (23.0)	1	1
Education level	Cannot read and write	45 (17.0)	54 (20.0)	0.86 (0.53–1.42)	1.58 (0.32–2.71)
	Can read and write	83 (31.0)	86 (32.0)	1	1
Marital status	Single	63 (23.5)	77 (28.7)	0.79 (0.49–1.28)	0.48 (0.23–1.12)
	In union	65 (24.3)	63 (23.5)	1	1
Prior TB history	Yes	24 (9.0)	30 (11.2)	0.85 (0.46–1.54)	0.52 (0.22–1.29)
	No	110 (41.0)	104 (38.8)	1	1
Body mass index	≤ 18 kg/m^2^	101 (37.7)	87 (32.5)	2.30 (1.32–3.93)	2.01 (1.31–3.85)
	> 18 kg/m^2^	27 (10.1)	53 (19.7)	1	1
Duration of cough	≤ 14 days	28 (10.4)	23 (8.6)	1.42 (0.77–2.63)	0.86 (0.58–2.15)
	> 14 days	100 (37.3)	117 (43.7)	1	1
Smear grading	< 2+	68 (25.4)	98 (36.6)	0.49 (0.29–0.81)	0.46 (0.21–0.73)
	≥ 2+	60 (22.4)	42 (15.7)	1	1
HIV status	Positive	27 (10.1)	13 (4.8)	2.61 (1.28–5.32)	2.38 (1.20–4.91)
	Negative	101 (37.7)	127 (47.4)	1	1
Having DM	Yes	31 (11.5)	17 (6.3)	2.31 (1.21–4.42)	2.11 (1.18–4.20)
	No	97 (36.2)	123 (46.0)	1	1
Knowledge on TB	Good	42 (15.7)	71 (26.5)	0.47 (0.29–0.78)	0.44 (0.23–0.69)
	Poor	86 (32.0)	69 (25.8)	1	1
Cigarette smoking	Yes	34 (12.7)	20 (7.5)	2.20 (1.25–4.01)	1.96 (1.20–3.82)
	No	94 (35.0)	120 (44.8)	1	1
Taking alcohol	Yes	34 (12.7)	36 (13.4)	1.05 (0.61–1.81)	0.82 (0.48–1.58)
	No	94 (35.1)	104 (38.8)	1	1
Faced stigma	Yes	61 (22.7)	41 (15.3)	2.20 (1.33–3.64)	2.0 (1.26–3.52)
	No	67 (25.0)	99 (40.0)	1	1
Length of TB service delay	< 82 days	54 (20.0)	96 (36.0)	0.33 (0.21–0.55)	0.35 (0.23–0.64)
	≥ 82 days	74 (27.6)	44 (16.4)	1	1

## Discussion

Sputum smear negativity (become non-infectious) after a certain period of anti-TB treatment initiation is an important marker to evaluate the response of anti-TB treatment in developing countries. Various literature showed different lengths of sputum and culture conversion time [20, 22]. This study revealed a median sputum smear conversion time of 35 days, IQR: 21–56 days. This was in line with former studies that reported a median time of 35 days [28] and 34 days with an IQR of 28–54 [23]. Alternatively, this finding was lower compared to a study finding from Afghanistan [20] where the sputum smear conversion time was 60 days. The observed discrepancy might be related to variations in the study period, socio-economic status of patients, and strength of TB control programs. The current finding was also found to be higher compared to findings of previous studies elsewhere; median time of 24 days [22] and 21 days [29]. The discrepancy might be related to the difference in length of the follow-up period to estimate the infectious period, characteristics of PTB cases (personal behaviors, nutritional status, and adherence to anti-TB drugs), and clinical profile of PTB cases including comorbidity status (HIV and DM). All these can determine the size of period of non-infectious after treatment initiation.

In most studies, the proportion of sputum smear conversion at the end of the 5th treatment was consistent with our findings [22, 23, 28]. Similarly, the proportion of smear conversion at the end of the 2nd month of anti-TB treatment (85%) was found to be in line with findings of former studies from Ethiopia [23], Italy [22], and Iran [30] where the proportions of sputum smear conversion were (81.6%), (78.7%) and 83.6% at the end of 2nd-month treatment, respectively. However, it was lower than study findings reported from Cameroon where proportions of sputum smear conversions were 93.4% [31] and Morocco [32] where 95% sputum smear conversion was reported by the end of the 2nd-month treatment follow-up period. Differences in the socio-economic status of TB patients, quality of TB monitoring strategies, comorbidity status, personal behaviors, and complications of illness might result in these discrepancies. In our study, the trend of sputum smear conversions showed a high increase between the end of the 1st month and 2nd months and a slight increase to the end of the 5th month of anti-TB treatment follow-up. This is possibly linked to the effectiveness of bacteriocidic anti-TB drugs given in the intensive phase that aimed to lower bacterial load [19].

Based on the multivariable logistic regression analysis, PTB cases who had ≤ 2+ sputum smear grades were 54% times less likely to have delayed sputum smear conversion compared to PTB cases with > 2+ smear grads. The possible explanation is linked to the high load of bacilli with PTB cases having higher smear grades that require time to be cleared and become zero compared to PTB patients with lower smear grades making other conditions constant. Prior studies also reported similar results [20, 22, 28].

The odds of having longer sputum smear conversion was twice among PTB cases who had HIV infection, and DM as comorbidity compared to the counterpart PTB cases. This was supported by previous studies from different parts of the world [20, 29, 30, 33]. This is attributed by diseases complications, unfavorable anti-TB treatment adherence, drug interactions, declined immunity status, undernutrition due to poor dietary intake, psychological stress, and societal stigma. All these can determine anti-TB treatment adherence. In addition, sputum conversion needs immune-competent PTB patients, and PTB patients with HIV, DM, and elders or children have weak immunity that lower sputum smear conversion rate [3]. This implies that timely integrated screening and management of comorbidities such as TB, HIV, and DM is required to improve the performance of TB prevention and control programs. This might prevent TB service delays, poor treatment outcomes, length of TB infectious period, and transmission to others.

Similarly, PTB patients with undernutrition were double times to have delayed sputum smear conversion compared to normal and overweight PTB patients. Former studies also reported undernutrition as a predictor variable to longer sputum smear conversion status [23, 29, 34, 35]. The possible explanation for this is due to the contribution of undernutrition to have weakened immunity status that makes people more susceptible to get TB infection, have high disease complications, and less chance of sputum smear conversion [3].

In this study, knowledge of PTB patients on TB was found to be a statistically significant factor in sputum smear conversion. Based on that, PTB patients having good knowledge were 56% times less likely to have delayed sputum smear conversion compared to PTB patients with good knowledge of TB. This might be linked to better anti-TB treatment adherence that people with better knowledge will do. If TB patients are aware of TB (prevention, diagnosis, and treatment), they will relatively have better TB service-seeking practice and adherence in anti-TB treatment follow-up [17]. This will then result in having non-delayed sputum smear conversion.

In addition, the smoking habit of PTB patients was reported as statistically significant with the length of sputum smear conversion. Thus, the odds of having long sputum smear conversion was twice among PTB patients who smoked cigarettes in any amount and frequency compared to non-smoker PTB patients. Previous studies on TB and smoking also reported supporting results [35–37]. This is attributed to the effect of smoking on lung defense mechanisms, lowering T-cell immunities, and poor dietary intake. These make people immune incompetent that makes them more susceptible to TB infection, high TB disease complications, and poor treatment adherence due to complications, drug–substance interactions, forgetfulness to take and collect anti-TB drugs [21, 37]. All these lead to have delayed sputum smear conversion and poor treatment outcomes including death.

Moreover, PTB patients that faced societal stigma had double odd of getting delayed sputum smear conversion than the counterpart PTB patients. Most people with less awareness about TB think that TB and HIV are inseparable. As a result, they have poor TB service-seeking practices and make stigmatization towards TB patients by assuming they have also HIV infection. This makes TB patients to be isolated from social events and friends. This results in getting poor supports, poor treatment adherence, psychological stress, and TB disease complications [15, 21]. Such conditions most likely end with poor sputum smear conversion time.

The length of TB service delay (patient and facility) also showed association with the length of sputum smear conversion. Consequently, PTB patients with lower TB service delay were 65% times less likely to have deferred sputum conversion than PTB patients with delayed TB service-seeking practices. Longer delay in seeking TB services results in disease complications, longer infectious period, and weakened immunity that might lead to other infections including HIV, DM, and undernutrition [7, 21, 38]. Thus, people who started anti-TB treatment after a longer TB service delay might have unfavorable treatment responses including deferred sputum conversion.

### Strength and limitations of the study

The strength of this study relies on employing the sputum concentration technique to increase the sensitivity of sputum smear detection. Estimating the proportions of sputum smear conversion at more points of treatment follow-up period might also be considered as a strength since it has a significant role in the monitoring of treatment follow-up periods. In this study, due to health education about TB and its treatment, and strong counseling and follow-up of TB cases, we make defaulting from anti-TB treatment zero with a 95% cure rate. The study also identified important variables that need to be considered to improve the status of treatment outcomes among PTB patients.

However, using sputum smear conversion due to the absence of sputum culture and other rapid TB diagnostic services might have little impact on estimating the size of proportions and median sputum smear conversion of PTB patients. Due to the absence of agreed-up cutoff value to group sputum smear conversion, I used a median value which might have some limitations. Thus, interpretation of findings needs to account for these limitations.

### Conclusions

Although the treatment success rate was better (95%), the median sputum smear conversion was higher compared to the TB program expectations and some former studies. Undernutrition, high prior smear grade, being HIV positive, having DM, poor knowledge on TB, cigarette smoking, facing stigma, and length of TB service delay were statistically significant factors to the length of sputum smear conversion. The highest effort is needed to improve the health literacy of the community by revising the existing community awareness strategies. This will improve their knowledge on TB, and shape their nutritional status, personal behaviors, and practicing societal stigma towards TB patients. Early management of comorbidities through offering integrated services for TB, HIV, and DM is required. A further study that considers large sample size and sputum culture is suggested to estimate the size of sputum smear conversion time.

## Supplementary Information

**Additional file 1.** Data set used to determine sputum smear conversion (contents of Figs. 1 and 2).

**Additional file 2.** Questionniare used to collect data in this research.

## Data Availability

The datasets used and/or analyzed during the current study are available from the corresponding author on reasonable request.

## Abbreviations

AFB: Ziehl–Neelsen acid-fast bacilli
AOR: Adjusted odds ratio
BMI: Body mass index
CI: The confidence interval
COR: Crude odds ratio
DM: Diabetes mellitus
HCs: Health centers
IQR: Inter-quartile range
MDR-TB: Multi-drug resistant tuberculosis
PHCU: Primary health care unit
PTB: Pulmonary tuberculosis
SD: Standard deviation, and smear-positive PTB
