# Supplementary material for: Sputum smear conversion and associated factors among smear-positive pulmonary tuberculosis patients in East Gojjam Zone, Northwest Ethiopia: a longitudinal study
Source: BMC Pulm Med. 2021 Apr 8;21:118. doi: 10.1186/s12890-021-01483-w (PMC8033743; doi:10.1186/s12890-021-01483-w)
Supplement: Supplementary file 1 — Additional file 1. Data set used to determine sputum smear conversion (contents of Figs. 1 and 2). [file 12890_2021_1483_MOESM1_ESM.docx]

1. **Questionnaire**

Determining the magnitude of TB infectious period after treatment initiation among PTB patients attending their anti-TB treatment.

**Patient interview questionnaire among PTB patients (new smear-positive and new smear-negative): put ‘√’ or ‘X’ in the boxes that you want to tick to indicate your answer.**

Questionnaire number____________ date of interview_____

Name of health facility_______________ Name of interviewer _________________

| No. | Questions | Responses | | Remark |
| --- | --- | --- | --- | --- |
| 1 | Age in years | ______________ | |  |
| 2 | Sex | Male Female | |  |
| 3 | Residence | Rural  urban | |  |
| 4 | Education level | unable to read and write  able to read and write | |  |
| 5 | Marital status | single  in union | |  |
| 6 | Had previous history of TB | yes No | |  |
| 7 | Prior sputum smear grading | scanty  +1  +2  +3 | |  |
| 8 | Distance from facility | ___________kilometers | |  |
| II | **Suggestive TB symptoms (Mark ‘‘X’’ in front of patient response)** | | | |
| 9 | Do you have a cough? | Yes No |  | |
| 10 | Do you have sputum? | Yes No |  | |
| 11 | If the patient’s answer is yes for question number #10 please ask if there is blood in the sputum. | Yes No |  | |
| 12 | Do you have shortness of breath? | Yes No |  | |
| 13 | Do you have chest pain? | Yes No |  | |
| 14 | Do you have a fever? | Yes No |  | |
| 15 | Do you have bodyweight loss? | Yes No |  | |
| 16 | Do you have night sweats? | Yes No |  | |
| 17 | Do you have a loss of appetite? | Yes No |  | |
| 18 | When did your cough/current symptoms start? | _______/_______/_____ | write (the date/ month/year) | |
| 19 | Formal healthcare provider the patient first visited after the onset of cough/current symptoms? | Health post  Health center  Public hospital  Private clinic  Private Hospital |  | |
| III | **Time of diagnosis and treatment start** | | | |
| 20 | When did you first visit a formal health care provider for the current illness? | _____/____/_______ | (write date/ month/year) | |
| 21 | When did you first diagnosed with TB at this health facility? | ____/______/_______ | (write date/ month/year) | |
| 22 | When did you first initiation of anti-TB drugs at the current health facility | _____/______/______ | (Write date/ month/year) | |
| 23 | Form of TB the patient has: | Smear positive pulmonary  Smear negative pulmonary |  | |
| 24 | Did you face stigma from any person or group because of your TB disease? | Yes No |  | |
| 25 | If yes for Question # 24, from whom? | community friends families  neighbors,  others _____ | Choose all possible | |
| 26 | What actions you took to mitigate the impact of stigma? | ____________________ |  | |
| 27 | Weight in KG | ____________________ |  | |
| 28 | Height in meter | ____________ |  | |
| 29 | Body mass index (BMI) | _________________ |  | |
| 30 | HIV tested | Yes  No  Unknown |  | |
| 31 | HIV testing result | Positive  Negative  Unknown |  | |
| 32 | Comorbidity that you have | ___________________ |  | |
| 33 | Do you have history of cigarette smoking in any amount and frequency? | Yes No |  | |
| 34 | Do you have history of taking alcohol in any amount and frequency? | Yes No |  | |

Note: some variables such as knowledge about TB, length of service delay, etc are composite variables derived from the given variables.

1. Sample format for reporting sputum smear follow up results of PTB cases for weekly manner.

| Ser.No | PTB patient ID | Weak and sputum smear result (positive /+ve/ or negative/-ve/) |
| --- | --- | --- |
| 1 |  |  |
| 2 |  |  |
| 3 |  |  |
| . |  |  |
| . |  |  |
| 268 |  |  |
